# A Nomogram Combining Two Novel Biomarkers for Predicting Lung Adenocarcinoma in Ground-Glass Nodule Patients

**DOI:** 10.1155/humu/8647969

**Published:** 2025-06-11

**Authors:** Yameng Li, Qingxian Zhang

**Affiliations:** Department of General Diseases, The First Affiliated Hospital of Zhengzhou University, Zhengzhou, China

**Keywords:** lung adenocarcinoma, nomogram, pulmonary ground-glass nodules, RNA sequencing technology

## Abstract

**Objective:** Combination of CT imaging and RNA sequencing techniques was used to explore the potential biomarkers specific to lung adenocarcinoma within pulmonary ground-glass nodules.

**Method:** The imaging and pathological data of patients with pulmonary ground-glass nodules who underwent chest CT scanning were confirmed through surgical procedures. Based on the pathological results, the patients were categorized into a benign nodule group and a malignant nodule group. Subsequently, RNA sequencing was conducted to analyze gene expression information in the pulmonary ground-glass nodules of these 16 patients.

**Results:** CT signs demonstrated statistical significance in both benign and malignant nodules. A total of 2080 upregulated genes and 1240 downregulated genes were identified through RNA sequencing in malignant nodules compared to benign nodules. CST1 exhibited increased expression among the upregulated genes in lung adenocarcinoma tissues compared to lung tissues. Among the downregulated genes, only GIMAP1-GIMAP5 showed decreased expression in lung adenocarcinoma tissues. Finally, we validated the clinical significance of CST1 and GIMAP1-GIMAP5 in patients with lung adenocarcinoma, particularly highlighting a strong correlation between GIMAP1-GIMAP5 expression levels and prognosis for patients. A visual nomogram predictive model for pulmonary ground-glass nodules was constructed (area under the receiver operating characteristic curve (AUC) > 0.8).

**Conclusion:** We constructed a nomogram combining CST1 and GIMAP1-GIMAP5 expression for predicting lung adenocarcinoma in ground-glass nodules in the context of COVID-19. This nomogram addresses the unique diagnostic challenges posed by COVID-19, where overlapping pulmonary imaging findings between viral pneumonia and early lung cancer necessitate robust molecular-aided discrimination.

## 1. Introduction

Lung cancer is the foremost cause of cancer-related mortality worldwide, with approximately 2 million new cases and 1.76 million deaths reported annually [1]. Due to the exorbitant cost associated with lung cancer diagnosis, most cases are already in advanced stages at the time of detection. Extensive efforts spanning four decades have been dedicated toward the early detection of lung cancer for prompt treatment initiation. While chest radiography and sputum screening tests have proven ineffective in reducing lung cancer mortality rates, the utilization of multiple low-dose CT scans has shown promise in facilitating early detection and screening [2]. In its initial stages, pulmonary glass nodules can be identified through CT imaging [3]. Pulmonary ground-glass nodules (GGNs) are characterized by nodules with well-defined or indistinct boundaries and localized, thin, and denser shadows observed in CT or X-ray scans of the lungs, revealing distorted trachea, vascular bundles, and interlobular septa. Previous studies have reported their volume doubling time ranging from 769 to 1005 days [4, 5]. Histopathologically, increased local lung tissue density is attributed to tumor infiltration, inflammatory cell aggregation, and alveolar cavity fluid accumulation leading to reduced gas content within each pixel unit and consequently higher CT values that manifest as GGNs on imaging. In this retrospective analysis of 16 patients with pulmonary GGNs conducted herein, we aim to provide a comparative analysis of CT imaging features and pathological findings in order to enhance the diagnostic accuracy for diseases associated with pulmonary GGNs while offering clinical guidance.

COVID-19, also known as coronavirus disease 2019, is a pandemic caused by the SARS-CoV-2 virus and is characterized by rapid transmission and widespread impact. Clinical manifestations of COVID-19 range from asymptomatic infection to mild upper respiratory tract disease and can progress to pulmonary involvement, hypoxemia, multiorgan dysfunction, shock, and death [6]. Studies suggest that the impact of COVID-19 on the immune system may exacerbate the condition of lung cancer patients, who are at a higher risk of severe illness and mortality [7]. Timely and accurate diagnosis of lung cancer is crucial for improving the prognosis of patients with COVID-19. Furthermore, the COVID-19 pandemic has disrupted the standard treatment regimens for numerous cancer patients, which may result in postponed lung cancer therapies. Such disruptions not only compromise the efficacy of cancer management but also potentially heighten the vulnerability of these patients to COVID-19 infections [8]. Therefore, early screening and diagnosis of lung cancer are particularly important during the COVID-19 pandemic. Research indicates that lung cancer patients incidentally identified during COVID-19 screening are often in the early stages, implying that early diagnosis can significantly improve treatment outcomes [9]. Most prior research on pulmonary GGNs has focused on non-COVID populations, where the challenge of differentiating malignant nodules does not involve overlapping imaging features with viral pneumonia. In contrast, this study addresses the unique scenario of COVID-19, where CT manifestations of GGNs in lung adenocarcinoma may resemble COVID-19-induced ground-glass opacities, necessitating molecular biomarkers to improve diagnostic accuracy during the pandemic. In conclusion, differential diagnosis between COVID-19 and lung cancer is of significant importance among patients with COVID-19.

With the rapid advancement of RNA sequencing technology, it has emerged as a potent tool for analyzing differentially expressed genes (DEGs). In cancer research, single-cell RNA sequencing enables comprehensive transcriptomic analysis at the resolution of individual cells, offering great potential for studying immune heterogeneity in the complex tumor microenvironment [10]. RNA sequencing can accurately identify tumor cell types, determine tumor grade and invasion degree, predict patient prognosis, and facilitate personalized treatment planning based on its results [11]. Numerous studies have demonstrated the widespread utilization of RNA sequencing technology in lung adenocarcinoma research. On one hand, this technology has unveiled the regulatory relationship between epithelial–mesenchymal transition and ferroptosis in lung adenocarcinoma [12], while also establishing and validating prognostic models for patients [13]. Furthermore, investigating B-cell marker gene characteristics in lung adenocarcinoma can aid in predicting prognosis and response to immunotherapy [14].

With the widespread application of chest CT in lung cancer screening, there has been an increasing prevalence of early lung adenocarcinoma presenting as pulmonary GGNs [15]. However, to date, no studies have investigated the genomic expression differences between benign and malignant nodules, and it remains unknown whether DEGs between these two types can serve as biomarkers for diagnosing and prognosticating lung adenocarcinoma. In this study, we utilized RNA sequencing technology to detect gene expression in eight benign nodules and eight malignant nodules, followed by DEG analysis. The functional annotation and signaling pathway analysis of differentially expressed genes were performed using a combination of GO database and KEGG database. Additionally, a search on the GEPIA database helped us predict potential genes involved in the progression of lung adenocarcinoma within GGNs, with the aim to provide guidance for diagnosis and prognosis by detecting specific gene expressions. Through rigorous differential expression analysis and validation in external datasets (GSE118370, GSE140343), we identified CST1 (upregulated in malignant nodules) and GIMAP1-GIMAP5 (downregulated in malignant nodules) as key candidates. CST1 has been linked to cancer-related pathways, while GIMAP1-GIMAP5 showed a strong association with patient survival outcomes, making them critical targets for investigating diagnostic and prognostic utility in this context.

## 2. Materials and Methods

### 2.1. Requisition of Clinical Data From Patients

**Figure 1:**
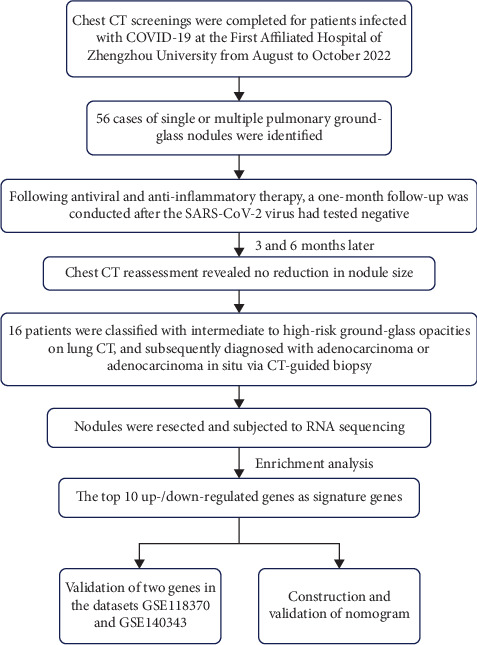
Study flowchart.

From August to October 2022, 16 COVID-19 patients with pulmonary GGNs treated surgically at Zhengzhou University's First Affiliated Hospital were analyzed. Detailed information is shown in Figure 1. The group included 8 males and 8 females, aged 27–79. Pathology divided them into benign and malignant nodule categories. COVID-19 diagnosis was confirmed by reverse transcription–polymerase chain reaction (RT-PCR) of nasopharyngeal swabs. Patients in both benign and malignant nodule groups were matched for COVID-19 severity (as defined by WHO clinical progression scale) and symptom duration to ensure comparability between groups. The study was approved by the Ethics Committee of the First Affiliated Hospital of Zhengzhou University (No. 2023-KY-0366) and was carried out according to the guidelines of the Declaration of Helsinki. Written informed consents were obtained from all patients.

Inclusion criteria for patient selection included histologically confirmed GGNs and available CT scans. This study focused exclusively on COVID-19 patients with GGNs to address pandemic-specific diagnostic challenges; thus, a non-COVID-19 GGN control group was not included in the current analysis. Exclusions were lung metastases, diffuse/transient nodules, poor lung function affecting image quality, and patients unwilling or unfit for surgery.

### 2.2. CT Scan

Patients received chest CT scans using GE Lightspeed 16-slice, GE Brightspeed 16-slice, or SIEMENS Sensation Cardiac 64-slice machines in a supine position, covering from lung apex to subpleural angle, including chest wall and axilla. Parameters are as follows: 120 kV, 250 mA, 512 × 512 matrix, 5 mm thickness, 5 mm spacing, 1 mm layer spacing, and 1.25 mm reconstruction layer.

### 2.3. Image Analysis

Image analysis focused on nodule size, location, density, internal features (e.g., empty spaces and air bronchograms), edge characteristics (lobulation and spiculation), lung-tumor interface clarity, and adjacent structures (pleural indentation and vascular convergence).

### 2.4. Histopathological Examination

Lung tissue was paraffin embedded, sectioned at 5 *μ*m, dewaxed, hydrated, and stained with hematoxylin for 5 min, 1% ethanol hydrochloride, and 0.5% eosin for 1 min. After ethanol dehydration and xylene washing, sections were mounted with neutral glue and observed under an Olympus microscope.

Following the 2011 lung adenocarcinoma classification, subtypes include the following: atypical adenomatous hyperplasia (AAH) with mild/moderate atypia without inflammation or fibrosis; adenocarcinoma in situ (AIS), ≤ 3.0 cm, with alveolar wall growth without infiltration; minimally invasive adenocarcinoma (MIA), ≤ 0.5 cm infiltrative growth within ≤ 3.0 cm lesions; and invasive pulmonary adenocarcinoma (IPA), with > 0.5 cm infiltration in ≤ 3.0 cm lesions. AAH and AIS are considered glandular precursors.

### 2.5. Observation Indicators

Observation indicators are as follows: (1) pathological diagnoses and (2) CT findings.

### 2.6. RNA Sequencing

RNA sequencing was performed on RNA from benign and malignant nodules after rRNA depletion, using the VAHTS V6 RNA sequencing Kit. cDNA was made, double-stranded, repaired, tailed, and linked. Purified fragments were PCR amplified for Illumina Hiseq 2500 sequencing in pair-end mode (150 bp reads). Clean reads were quality-checked with Fast-QC and mapped to a reference genome for gene counting (FPKM normalization). DEGs were identified by DESeq2 (Log2FC > 1 or < −1, FDR < 0.05), followed by GO and KEGG analyses on the KOBAS 2.0 server.

### 2.7. Bioinformatics Analysis

GEPIA (http://gepia.cancer-pku.cn/) analyzes RNA sequencing data from 9736 tumors and 8578 normals in TCGA and GTEx, offering differential expression, cancer-specific, survival, gene similarity, correlation, and dimensionality reduction analyses, with GTEx and TCGA data for gene testing. We also conducted an analysis of the GSE118370 and GSE140343 datasets to investigate the differential gene expression between tumor and normal tissues using GEO2R.

### 2.8. Statistical Analysis

The statistical analysis was conducted using SPSS 20.0 software, while the qualitative data were analyzed employing either the chi-square test or Fisher's exact probability method.

Nomograms (a graphical tool used to estimate the probability of a specific outcome based on multiple input variables) assigned a score to each predictor based on its contribution to the outcome variable within the predictive model. The total score was calculated by summing the individual scores of each predictor, and the probability of central precocious puberty was derived from the total score scale. Furthermore, the performance of the predictive model was assessed using the AUC, the precision–recall (PR) curve.

## 3. Results

### 3.1. General Information and CT Signs of Patients Presenting With Diverse Histopathological Subtypes of Pulmonary GGNs

Of 16 patients, 8 males and 8 females aged 36–79 were studied, with 8 benign and 8 malignant nodules identified. The malignant group included 3 AIS, 3 MIA, and 2 IPA cases. No age or sex differences were found between benign nodules and the three malignant types.

Of 8 malignant nodule patients, 4 had left lung lesions, 2 had right lung lesions, and 2 were bilateral. No significant location difference existed between benign and malignant cases. Larger nodules correlated with more invasive lung adenocarcinoma (Table 1).

### 3.2. Typical Imaging-Pathological Data Presentation

**Figure 2:**
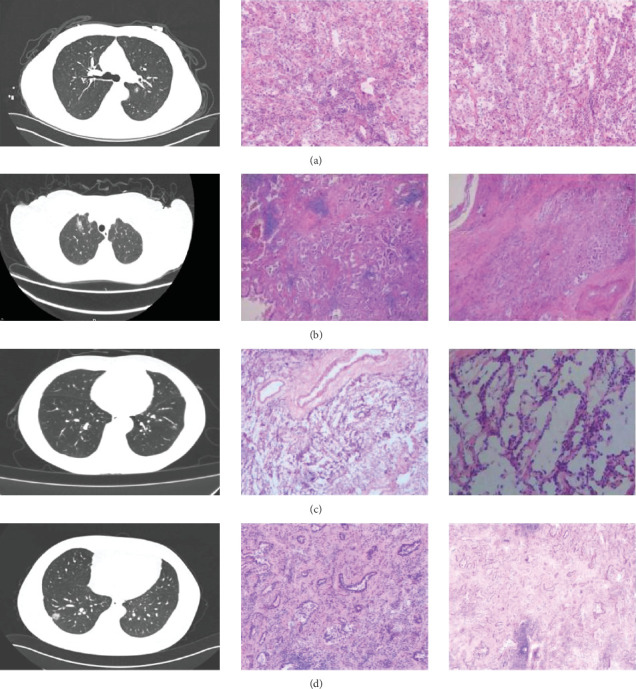
Typical imaging-pathological data presentation. (a) Benign nodule, (b) AIS, (c) MIA, and (d) IPA.

CT showed a 1.4∗1.1∗0.6 cm benign nodule in the left upper lobe with medium density and indistinct margins, surrounded by gray-red, soft lung tissue with chronic inflammation and fibrous tissue hyperplasia, and signs of organizing pneumonia. No abnormalities were seen in bronchial or vascular stumps, leading to a pneumonic pseudo tumor diagnosis (Figure 2a). AIS CT showed a 30-mm high-density nodule in the right upper lobe, pathologically a moderately differentiated adenocarcinoma, 3.0∗1.5∗1.0 cm, without airway spread, thrombus, invasion, or lymph node metastasis (Figure 2b). MIA CT revealed an 8.6∗5.7∗2.0 cm nodule in the left lower lobe without pleural invasion or bronchial or portal vein involvement (Figure 2c). IPA CT showed GGNs and a 15-mm nodule in the right upper lobe, with pathology of moderately differentiated acinar, adherent, and papillary adenocarcinoma, no cancer in stumps or margins, and no pleural invasion on elastic fiber staining (Figure 2d).

### 3.3. The Identification of Genes With Differential Expression Between Benign and Malignant Nodules

**Figure 3:**
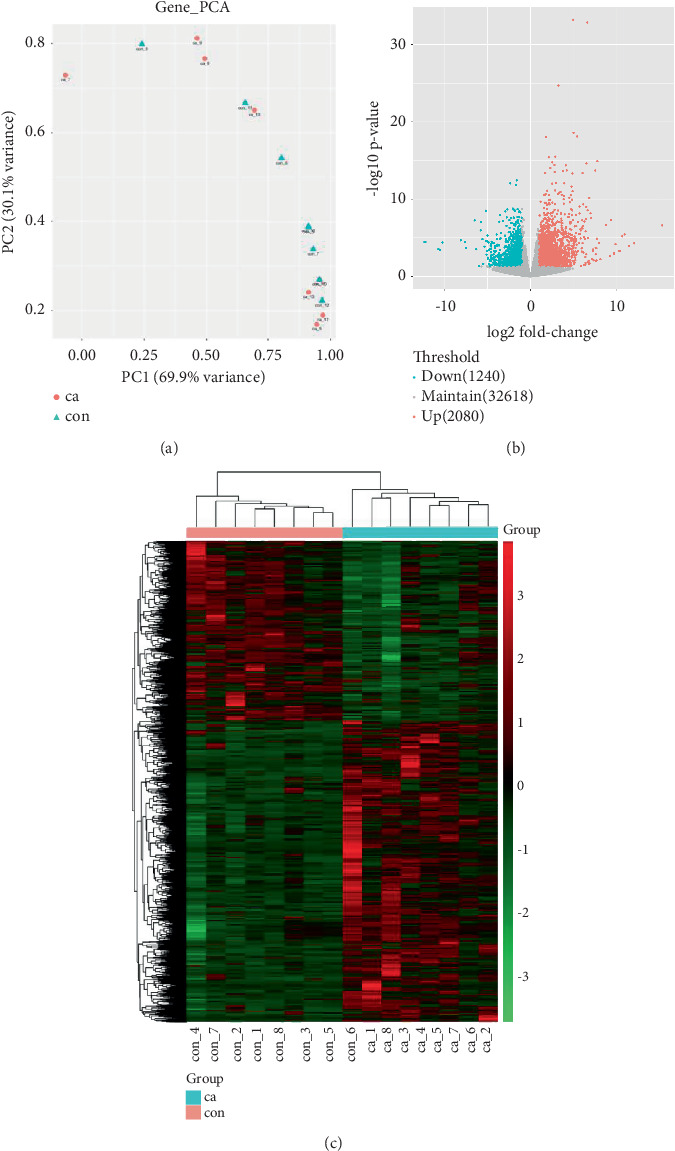
The differential expression of genes between benign and malignant nodules. (a) Principal component analysis. (b) Volcano plot displaying the fold change and *q* value of coding genes between benign and malignant nodules. The *x*-axis represents the fold change in gene expression, which is considered biologically significant only when the threshold of change is set at Log2FC > 1 or < −1. The *y*-axis represents statistical significance, with a threshold set at a *q* value of 0.05 (−Log10*q* value > 1.3). Upregulated genes, indicated by red dots, have a fold change greater than 2 and *p* value less than 0.05. Downregulated genes, shown as green dots, have a fold change less than 0.5 and *p* value less than 0.05; genes with nonsignificant differences are represented by gray dots. (c) Clustering analysis of differentially expressed genes: The *x*-axis represents sample names between groups, while the *y*-axis represents differentially expressed genes. In this figure, high expression values of differentially expressed genes in group samples are denoted by red dots, whereas low expression values are denoted by green dots.

The gene expression profile of all samples was analyzed using principal component analysis (PCA), which revealed a clear separation between benign and malignant nodules (Figure 3a). To identify potential DEGs between the two groups, we conducted DEG analysis on 35,938 genes through sequencing analysis. As a result, we identified 2080 upregulated genes and 1240 downregulated genes in malignant nodules compared to the benign nodule group (Figure 3b,c).

### 3.4. Enhanced Analysis of the Function and Signaling Pathway of DEGs Between Benign and Malignant Nodules

**Figure 4:**
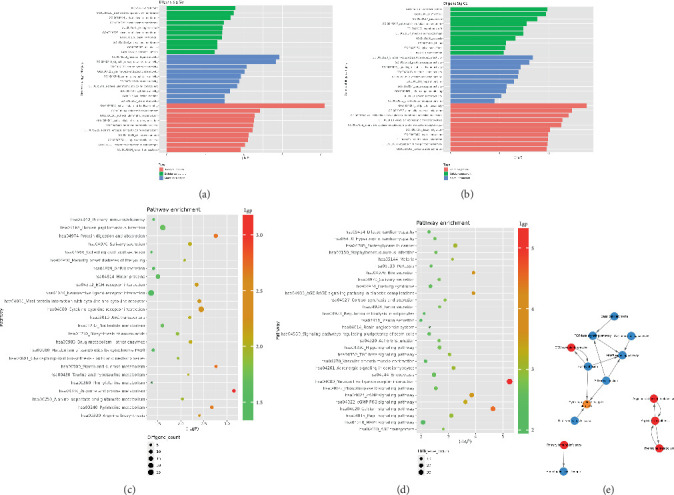
Functional and signaling pathway analysis of significantly differentially expressed genes. (a) Functional histogram of significantly upregulated differentially expressed genes. (b) Functional histogram of significantly downregulated differentially expressed genes. In the figure, the vertical axis represents the functional names of differentially expressed genes, and the horizontal axis represents the negative logarithm of *p* value (−LgP). A higher value indicates a smaller *p* value, indicating a greater significance level for the function of differentially expressed genes (only displaying the top 10 items in descending order based on −LgP values). (c) Bubble chart illustrating significant pathways enriched with upregulated differentially expressed genes. (d) Bubble chart illustrating significant pathways enriched with downregulated differentially expressed genes. In the figure, the vertical axis represents the pathway names for differentially expressed genes, and the horizontal axis represents the negative logarithm of p value (−LgP). A higher value indicates a smaller *p* value, indicating a greater significance level for that particular pathway. The size of each bubble corresponds to the number of differentially expressed genes within that pathway. (e) Interaction network diagram depicting significant pathways. In this diagram, circles represent individual pathways while straight lines indicate interactions between them. Red circles represent pathways associated with upregulated gene expression, blue circles represent pathways associated with downregulated gene expression, and yellow circles represent pathways exhibiting both up and downregulation properties.

The GO database analyzed gene and protein functions in pulmonary GGNs, highlighting upregulated genes in membrane-related functions like receptor activity and processes such as extracellular matrix degradation (GO:0022167), epidermal development (GO:0008544), extracellular matrix organization (GO:0030198), extracellular structure reconstruction (GO:0043062), and hormone metabolism (GO:0042445). Downregulated genes were linked to cell junction assembly (GO:0034329), cell migration (GO:0001667), and cell adhesion (GO:0098742) (Figure 4a,b). KEGG pathway analysis using Fisher's and chi-square tests identified significant pathways with *p* < 0.05, showing upregulated genes in arginine and proline metabolism, protein digestion and absorption, starch and sucrose metabolism pathways, and downregulated in neuroactive ligand–receptor interaction and calcium and cAMP signaling pathways, all related to cancer (Figure 4c,d).  Figure 4e shows an interaction network of these pathways.

### 3.5. GIMAP1-GIMAP5 in Lung GGNs Can Serve as Biomarkers for the Detection of Lung Adenocarcinoma

**Figure 5:**
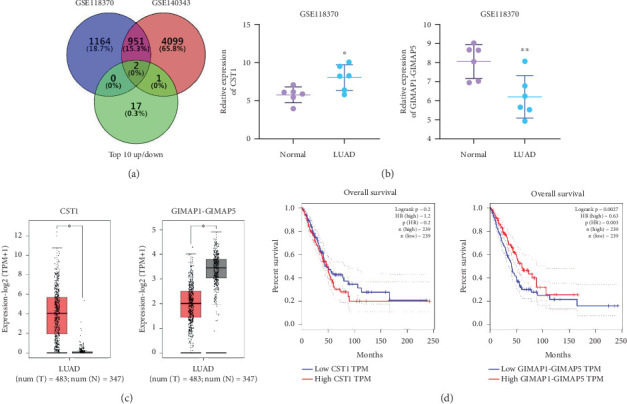
Expression patterns of CST1 and GIMAP1-GIMAP5 in lung adenocarcinoma and their prognostic implications. (a) GSE118370 and GSE140343 datasets were selected for including gene expression profiles of lung adenocarcinoma tissues and normal lung tissues to validate differentially expressed genes identified in our study. The Venn diagram illustrates the intersection of datasets GSE118370, GSE140343, and the Top 10 upregulated or downregulated genes. (b) Within the GSE118370 dataset, expression of CST1 and GIMAP1-GIMAP5 in lung cancer tissues. (c) Expression of the CST1 gene and GIMAP1-GIMAP5 gene in lung adenocarcinoma tissues. (d) Correlation between the expression level of CST1, GIMAP1-GIMAP5 and the prognosis of patients with lung adenocarcinoma.

Using GEPIA, we analyzed the Top 10 upregulated genes (PRSS2, AC093525.2, FAM157B, CST1, TBC1D3B, CXorf49B, C7orf55-LUC7L2, AC008758.3, AD000671.1, and CXorf49) and downregulated genes (CHURC1-FNTB, BLOC1S5-TXNDC5, TMEM189-UBE2V1, TBC1D3D, AC091304.1, AL136295.4, AL158066.1, GIMAP1-GIMAP5, MKX, and TRDN) in lung adenocarcinoma versus normal lung tissues (Table 2). Upon intersecting with the datasets GSE118370 and GSE140343, we identified that CST1 and GIMAP1-GIMAP5 were the only genes exhibiting significant differential expression in lung adenocarcinoma tissue compared to normal tissue (Figure 5a). Specifically, CST1 was upregulated, whereas GIMAP1-GIMAP5 was downregulated in lung adenocarcinoma tissue when contrasted with normal tissue (Figure 5b). These results were consistent with those obtained from the GEPIA database (Figure 5c). Survival analysis conducted using the GEPIA database revealed no significant correlation between the expression levels of CST1 and the survival rates of lung adenocarcinoma patients. However, an upward trend in survival rates was observed between the 50th and 150th months postdiagnosis. In contrast, low expression of GIMAP1-GIMAP5 was associated with shorter survival times (*p* = 0.0027), with a relative risk of 0.63 (*p* = 0.004), suggesting its potential prognostic value (Figure 5d).

### 3.6. Development of a Predictive Nomogram Model for Lung Cancer

**Figure 6:**
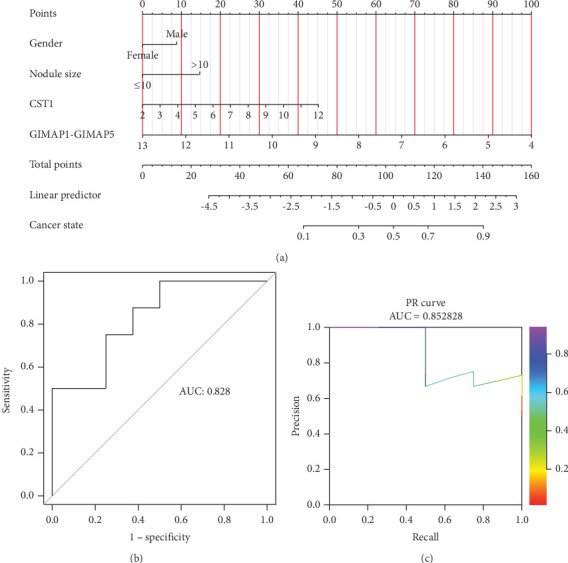
Lung cancer risk nomogram predictive chart. (a) Predictive nomogram model incorporating five variables (gender, nodule size, CST1, and GIMAP1-GIMAP5) for estimating the probability of lung cancer using a binary logistic regression framework. (b) Receiver operating characteristic (ROC) curve of the binary logistic regression model developed with gender, nodule size, CST1, and GIMAP1-GIMAP5 to predict lung cancer probability. (c) Precision–recall (PR) curve of the prediction model integrating gender, nodule size, CST1, and GIMAP1-GIMAP5 to assess the likelihood of lung cancer.

Incorporating five predictive variables—gender, nodule size, CST1, and GIMAP1-GIMAP5—into a binary logistic regression model, we aimed to predict the probability of lung cancer. The predictive probability was calculated using the following formula: log[*P*/(1 − *P*)] = 2.776 + 0.52(Gender_Male_) − 0.867(Nodule.size_≤10_) + 0.268(CST1) − 0.659(GIMAP1 − GIMAP5). The outcomes of the predictive nomogram model are illustrated in Figure 6a. According to the ROC curve, the AUC value of the prediction model was 0.828, indicating good discrimination (Figure 6b). Additionally, the PR curve, depicted in Figure 6c, exhibits an AUC value of 0.852828, underscoring the model's superior accuracy and efficacy.

## 4. Discussion

Significant advancements have been achieved in the field of lung cancer imaging, encompassing screening, diagnosis, differential diagnosis, efficacy evaluation, prognosis, and recurrence prediction [16–18]. The widespread utilization of low-dose CT scans for lung cancer has resulted in a substantial increase in the detection rate of pulmonary nodules. Furthermore, the persistent presence of pulmonary GGNs is indicative of the occurrence of lung adenocarcinoma [19]. Studies have reported that subjects undergoing low-dose CT examinations experience a 15%–20% reduction in annual lung cancer mortality rates compared to those who undergo chest x-ray examinations [20]. In lung cancer screening experiments, approximately half of the subjects exhibit pulmonary nodules; however, the vast majority are benign [21, 22]. Lung cancer screening's main challenge is early nodule differentiation. CT scans are crucial for identifying benign and malignant nodules by showing their relationships with vessels and bronchi. Key imaging indicators are nodule size, density, morphology, and signs like vacuole and vascular clusters. These indicators vary with nodule pathology and are essential for diagnosing and staging lung adenocarcinoma [23]. Hu et al. discovered that 56% of pulmonary nodules with a diameter of ≤ 10 mm exhibited malignancy, while 88.7% of pulmonary nodules with a diameter of ≥ 10 mm were found to be malignant [24]. Previous studies have demonstrated a positive correlation between nodule diameter and likelihood of lung cancer development. It is demonstrated that pulmonary GGNs displaying lobulation and spiculation displayed a higher degree of malignant infiltration compared to those without lobulation and smooth margins, indicating that the presence of lobulation and spiculation could serve as predictive indicators for the extent of pulmonary adenocarcinoma infiltration [23]. In this study, we retrospectively analyzed CT imaging and pathology of GGNs in the lungs of 16 patients with COVID-19. The findings revealed that malignant nodules exhibited significantly larger sizes compared to benign nodules, and the size of malignant nodules demonstrated a positive correlation with the extent of malignancy in lung adenocarcinoma.

However, it is important to note that the precision of CT scans might be compromised during the COVID-19 pandemic. The primary diagnostic modality for COVID-19 is chest CT scanning, which boasts a high sensitivity of up to 97%, which indicates that CT scanning is an effective tool for detecting COVID-19 [25, 26]. The hallmark CT manifestations of COVID-19, characterized by bilateral ground-glass opacities and areas of consolidation, may be subtle in the early stages of the disease but become increasingly evident as the condition advances. These radiological features parallel certain CT attributes of lung cancer, including ground-glass opacities and consolidations [27, 28]. Consequently, utilizing CT scans for lung cancer diagnosis amidst the COVID-19 pandemic presents significant challenges, given that COVID-19 infections can independently induce analogous radiological alterations in the pulmonary parenchyma. Furthermore, COVID-19 infections may inflict direct damage on lung tissues, encompassing alveolar injury and inflammatory responses, potentially exacerbating the CT manifestations [29]. Additionally, the inflammatory cascade triggered by COVID-19 could stimulate the proliferation of cancer cells and the reactivation of quiescent cancer cells, thereby impacting the tumor microenvironment [30, 31]. This suggests that COVID-19 infections might not only complicate the CT-based diagnosis of lung cancer but also influence its progression and clinical outcomes.

Transcriptomics, as the foremost and extensively utilized molecular technology in fundamental research, clinical diagnosis, and drug development [32], has been widely applied in cancer research through the help of RNA sequencing [33]. This study found 35,938 DEGs in lung GGNs, with 2080 up and 1240 downregulated in malignant nodules. GO analysis showed upregulated genes in processes like extracellular matrix degradation and hormone metabolism, while downregulated genes were linked to cell junction, migration, and adhesion. Furthermore, KEGG analysis demonstrated that the upregulated genes were enriched in arginine and proline metabolism pathways as well as protein digestion and absorption pathways, whereas the downregulated genes were mainly enriched in pathways such as neuroactive ligand-receptor interaction, calcium signaling, cAMP signaling, most of which are closely associated with cancer occurrence and progression.

The CST1 protein, belonging to the cystatin superfamily, acts as a potent inhibitor of cysteine proteases by binding to their active sites and inhibiting their hydrolytic activity [34]. While primarily distributed in the submandibular gland, gallbladder, and uterus, CST1 exhibits high expression levels in malignant tissues [35]. Numerous studies have implicated CST1 in the progression of various tumors, including gastric cancer, colorectal cancer, and breast cancer. It has also been identified as a prognostic indicator for cancer [36–38]. Interestingly, there is emerging evidence linking CST1 to lung cancer. Lai et al. demonstrated that serum CST1 can serve as a diagnostic marker for distinguishing early non-small cell lung cancer from benign lung nodules [39]. Recent research has further revealed that elevated expression of CST1 is associated with lung adenocarcinoma and promotes epithelial–mesenchymal transition. Moreover, it correlates with prognosis and tumor immune microenvironment in this context; thus, making it a potential prognostic biomarker for lung adenocarcinoma [40]. Our study noted higher CST1 expression in malignant nodules. Bioinformatics confirmed its upregulation in lung adenocarcinoma versus normal tissues. While overall survival did not differ by CST1 expression, a significant survival trend emerged between Months 50 and 150, suggesting CST1 in ground-glass opacities may be a lung adenocarcinoma biomarker. Notably, while a survival trend was observed for CST1 between 50 and 150 months postdiagnosis, this association did not reach statistical significance (*p* > 0.05), highlighting the need for caution in interpreting this finding and the importance of validation in larger datasets.

GIMAPs are nucleotide-binding proteins that belong to the immune-related nucleotide subfamily of the GTP-binding superfamily. Studies have linked GIMAPs to cancer. Bioinformatics show GIMAP1, GIMAP5, GIMAP6, GIMAP7, and GIMAP8 are downregulated in breast cancer. These findings suggest that these genes can serve as immune-related prognostic biomarkers within the tumor microenvironment of breast cancer [41]. Deng et al. also reported low expression levels of GIMAPs in lung adenocarcinoma compared to adjacent normal lung tissue [42]. Our study found lower GIMAP1-GIMAP5 expression in malignant nodules and linked it to shorter survival in lung adenocarcinoma patients, indicating its clinical importance for diagnosis and prognosis in this disease. Mechanistically, GIMAP proteins are part of the GTP-binding superfamily, implicated in immune cell homeostasis and activation [41]. Downregulation of GIMAP1-GIMAP5 may disrupt immune surveillance in the tumor microenvironment, potentially impairing T-cell function or promoting immune evasion, which could accelerate tumor progression and worsen prognosis. This aligns with prior research linking GIMAP family members to immune-related pathways in cancer [41, 42], suggesting a role in modulating antitumor immune responses.

Unlike prior studies, our comprehensive analysis of CT imaging and gene expression in GGNs, in the context of COVID-19, is aimed at identifying new lung adenocarcinoma genes. The retrospective design and small sample size (*n* = 16) restrict the generalizability of our findings. In this study, the nomogram was developed using a binary logistic regression model incorporating clinical and molecular variables. However, due to the retrospective design and relatively small sample size (*n* = 16), we were unable to report detailed regression coefficients for each variable or generate a calibration plot to assess model fit. These limitations highlight the exploratory nature of this work and the need for future validation in larger, prospective cohorts to refine the nomogram's predictive accuracy and statistical robustness. With no existing research on GIMAP1-GIMAP5's link to lung adenocarcinoma, our initial findings suggest the necessity for further studies to explore their role and impact on the disease's progression. A limitation of this study is the absence of a non-COVID-19 GGN control group, which prevents direct comparison of COVID-19-specific versus general GGN gene expression profiles. Future studies should include non-COVID-19 GGN cohorts to validate whether CST1 and GIMAP1-GIMAP5 serve as pandemic-unrelated biomarkers or are influenced by COVID-19-associated lung inflammation.

In the future, it will be essential to determine if the expression of GIMAP1-GIMAP5 is directly linked to COVID-19 infection or if this association is subject to the influence of other factors. This requires additional research and data analysis. For example, studies with larger cohorts are necessary to validate the initial observations and to investigate the expression patterns of GIMAP1-GIMAP5 at different stages of COVID-19 infection. Moreover, research should encompass longitudinal analyses of GIMAP1-GIMAP5 expression in patients with lung adenocarcinoma, both prior to and following COVID-19 infection, to establish whether the alterations in expression are a consequence of the virus's direct effects or are induced by the immune response it triggers.

In summary, CT imaging and RNA sequencing analysis of benign and malignant nodules, combined with a nomogram, suggest that CST1 and GIMAP1-GIMAP5 are potential biomarkers for lung adenocarcinoma of GGNs, providing new insights into its pathogenesis and treatment. Clinically, the nomogram could be incorporated into routine workflows by integrating it with liquid biopsies—noninvasive tests analyzing circulating tumor DNA (ctDNA) or microRNAs—to enhance diagnostic confidence, especially in COVID-19 patients with ambiguous GGN imaging. By combining molecular biomarkers (CST1 and GIMAP1-GIMAP5), clinical data, and CT characteristics, this tool offers a personalized risk stratification approach to guide decisions on surveillance, biopsy, or intervention, potentially minimizing unnecessary procedures and improving timely management of lung adenocarcinoma in pandemic contexts. This nomogram provides a valuable tool for differentiating lung adenocarcinoma from COVID-19-related pulmonary changes in GGNs, addressing the critical need for accurate diagnosis amidst pandemic-related imaging ambiguities.

## Figures and Tables

**Table 1:** General information and CT signs of COVID-19 patients with pulmonary ground-glass nodules.

	**Patient ID number**	**Age**	**Gender**	**Nodular location**	**Nodule size**	**Pathological diagnosis**
Benign nodule	con_1	37	Female	Inferior lobe of right lung	7 mm	Inflammation of the right lower lobe
	con_2	57	Male	Superior lobe of left lung	6 mm	Upper left inflammatory pseudotumor
	con_3	50	Female	Superior lobe of right lung	14 mm	Chronic inflammation of the right upper lung
	con_4	47	Male	Multiple pulmonary lesions	6 mm	Right upper alveolar epithelial hyperplasia
	con_5	36	Male	Multiple pulmonary lesions	14 mm	Inflammation of the right upper lobe
	con_6	53	Female	Superior lobe of left lung	12 mm	Inflammation of the left upper lobe
	con_7	52	Male	Superior lobe of left lung	3 mm	Inflammation of the left lower lobe
	con_8	38	Male	Superior lobe of left lung	5 mm	Chronic inflammation of left upper lung

Malignant nodule	ca_1	50	Female	Superior lobe of right lung	30 mm	AIS
	ca_2	56	Male	Inferior lobe of right lung	5 mm	MIA
	ca_3	64	Female	Left upper lung apex, left lower lung	12 mm	AIS
	ca_4	79	Female	Superior lobe of right lung	11 mm	AIS
	ca_5	54	Male	Inferior lobe of left lung	8 mm	MIA
	ca_6	75	Female	Inferior lobe of left lung	17 mm	MIA
	ca_7	59	Male	Inferior lobe of right lung; upper lobe of right lung and lower lobes of both lungs	9 mm	IPA
	ca_8	73	Female	Right lung apex, tongue segment of left lung and lower lobes of both lungs	15 mm	IPA

**Table 2:** Top 10 upregulated or downregulated differentially expressed genes in lung ground-glass nodules.

**Name of gene**	**Gene_id**	**Log2FoldChange**	**Gene location**
PRSS2	ENSG00000275896.5	15.36859197	Chr 7: 142760398-142774564
AC093525.2	ENSG00000260272.1	12.1137148	Chr16: 2496032-2520218
FAM157B	ENSG00000233013.10	11.47653061	Chr9: 138217068-138253217
CST1	ENSG00000170373.8	11.00609815	Chr20: 23747553-23751268
TBC1D3B	ENSG00000274808.5	10.71284634	Chr17: 36165681-36176636
CXorf49B	ENSG00000215113.6	10.43461618	ChrX: 71763424-71767204
C7orf55-LUC7L2	ENSG00000269955.2	10.33189855	Chr7: 139341360-139422599
AC008758.3	ENSG00000248406.1	9.932575486	Chr19: 12379746-12383687
AD000671.1	ENSG00000188223.9	9.777028277	Chr19: 35745678-35754519
CXorf49	ENSG00000215115.6	9.734100365	ChrX: 71714371-71718151
CHURC1-FNTB	ENSG00000125954.12	−12.35672336	Chr14: 64914485-65061803
BLOC1S5-TXNDC5	ENSG00000259040.5	−12.24590133	Chr6: 7881522-8064364
TMEM189-UBE2V1	ENSG00000124208.16	−10.74790035	Chr20: 50081124-50153637
TBC1D3D	ENSG00000274419.6	−10.5933422	Chr17: 38003976-38014902
AC091304.1	ENSG00000237850.7	−10.28823579	Chr15: 28349051-28358080
AL136295.4	ENSG00000259522.3	−8.1285166	Chr14: 24180395-24190416
AL158066.1	ENSG00000217576.7	−7.637766389	Chr13: 52167709-52291557
GIMAP1-GIMAP5	ENSG00000281887.3	−7.539645376	Chr7: 150716668-150743646
MKX	ENSG00000150051.13	−6.497791585	Chr10: 27672875-27746060
TRDN	ENSG00000186439.12	−6.310002543	Chr6: 123216339-123637093

## Data Availability

The data that support the findings of this study are available from the corresponding author upon reasonable request.

## Nomenclature

CT: computed tomography
AIS: adenocarcinoma in situ
MIA: minimally invasive adenocarcinoma
IPA: invasive pulmonary adenocarcinoma
DEG: differentially expressed gene
